# Development and Testing of the Thermoelectric Thermal Energy Conversion Device in the Conditions of Existing Aluminum Production

**DOI:** 10.3390/ma15238526

**Published:** 2022-11-30

**Authors:** Viktor V. Kondratiev, Ivan A. Sysoev, Aleksandr D. Kolosov, Vera V. Galishnikova, Vitaliy A. Gladkikh, Antonina I. Karlina, Yuliya I. Karlina

**Affiliations:** 1 Laboratory of Geochemistry of Ore Formation and Geochemical Methods of Prospecting, A. P. Vinogradov Institute of Geochemistry of the Siberian Branch of the Russian Academy of Sciences, 664033 Irkutsk, Russia; 2 LLC Scientific Research Center of Energy-Resource-Saving Technologies, 664074 Irkutsk, Russia; 3 JSC EuroSibEnergo, 664082 Irkutsk, Russia; 4 Stroytest Research and Testing Center, Moscow State University of Civil Engineering, 26, Yaroslavskoye Shosse, 129337 Moscow, Russia

**Keywords:** thermoelectric converter, energy saving, thermal power engineering, thermal energy utilization, heat exchange, gas flue

## Abstract

The aim of the work is to develop an energy-saving device that provides the conversion of thermal energy into electrical energy. The design and materials of the thermoelectric converter unit, consisting of 12 thermoelectric converter modules, a cooling radiator and a switching unit, were developed and selected. Based on the test results, the zone of the maximum temperatures in the section of the gas duct recommended for the installation of a gas cooling module using a thermoelectric converter was determined. The technology for cooling gases with the help of a thermoelectric converter was tested on the site located in front of the experimental heat exchanger. An assessment of the efficiency of the conversion of heat into electrical energy was conducted using the design of the thermoelectric converter unit, based on thermoelectric modules TGM 127-1.4-1.2. It was determined that the device is capable of generating electricity stably for production needs. The data obtained showed that, at a temperature difference of 75–80 °C between the wall surface of the gas duct section and the coolant, the power of one thermoelectric converter block of the gas cooling system reaches 9 W.

## 1. Introduction

At present, the issues involved in improving energy efficiency technologies in various fields, especially energy-intensive industries such as metallurgy, are becoming increasingly important, and this will continue in the foreseeable future [1,2,3,4,5,6,7]. The application of the Seebeck effect in strategic industries [8,9] is also of high importance for development, including that of space areas. One of the leading directions in the development of materials for TEC is skutterudites [10,11,12,13].

Heat exchange equipment, with its apparent simplicity, is the basis of a huge number of production processes [14]. Heat exchangers are used in the petrochemical, chemical and food industries and are of great importance for the thermal generation of electricity. Technologies for the design, construction and production of heat exchange equipment are developing at a rapid pace, providing an ever more impressive performance. One of the directions for the use of modern heat exchangers is the capture and utilization of waste heat from various industries. Such emissions occur in most large enterprises, from hydroelectric power plants and food production to metallurgical and machine-building plants. Waste heat, in addition to obvious energy losses, leads to the thermal pollution of the environment, which has a destructive effect on the fragile ecosystem of our planet.

In metallurgy, as well as other industries, production processes are associated with the release of hot exhaust gases. One of the most effective solutions for heat recovery is the use of heat exchangers [15,16,17,18,19,20,21]. Heat recovery, in addition to reducing thermal pollution and reducing energy losses, has very noticeable impacts on the economic and environmental sides of the production process. From the point of view of economics, the extraction of heat from the waste gases of metallurgical production significantly reduces the physical volume of gases. Reducing the physical volume of gas enables very significant savings in CAPEX and OPEX gas cleaning plants and gas removal systems. The environmental impact of the exhaust gas heat extraction aims to optimize the temperature regimes of the gas cleaning equipment. For example, the optimal temperature for the adsorption of hydrofluoride on alumina particles in dry gas cleaning systems for aluminum production is in the range of 60–80 °C. Without the use of a heat recovery system, the dust-gas mixture from modern high-current electrolyzers is fed into the gas cleaning system at a temperature that is too high, which significantly reduces the efficiency of the gas cleaning system.

With all the advantages of heat exchangers in reducing the temperature of flue gases and recovering waste heat, the energy received in the form of a coolant heated to a certain temperature does not always find proper application in real production conditions. In addition, there is always an interest among advanced enterprises in industrial energy harvesting. For example, gas duct routes connecting elements of heat exchangers (as in this work) are hot surfaces, and the heat they produce is insignificantly dissipated in the atmosphere. Heat recovery from these surfaces, with the additional cooling of the gas flow and generation of electrical energy for household enterprises, is an urgent task, one of the solutions to which is considered in this paper.

In the conditions of Siberia, the use of TEC technologies and heat exchangers was not previously introduced due to the low cost of electricity in this region. However, excessive thermal emissions from exhaust gases are a problem, not only because they lead to the overheating of industrial premises but also because they harm the environment. Therefore, in order to recover heat from the heated surfaces of gas ducts, it is recommended that one uses specially designed and mounted thermoelectric converters. The advantage of this technology is the possibility of converting thermal energy into electrical energy, as well as its direction for production needs [22,23]. An alternative option is the use of a heat exchanger. However, the possible use of hot water is much lower than the amount of energy that must be extracted from the exhaust gases of the RA-550 heavy-duty aluminum production technology. An analysis of existing solutions in the study area showed that the use of thermoelectric converters is a promising solution [24,25].

## 2. Materials and Methods

The decrease in the temperature of the electrolysis gases discharged by the gas removal system is provided using an experimental heat exchanger. This device allows one to significantly reduce the temperature of the exhaust gases without a significant increase in the aerodynamic resistance of the system. The heat exchanger is equipped with an automated monitoring system for the temperature at the inlet and outlet, pressure drop and gas humidity at the outlet of the heat exchanger. By reducing the exhaust gas temperature, it becomes possible to achieve a more optimal mode of operation of the gas cleaning system in terms of the cleaning efficiency, as well as the efficiency of the draft equipment. The energy of the exhaust gases is transferred to the coolant (water), after which it is used for the technological and household needs of the enterprise.

The design of the heat exchanger involves the additional utilization of waste heat, together with the production of electrical energy, by installing a system of thermoelectric converters on the transition cones of the heat exchanger. The electrical energy obtained in this way is used, for example, to illuminate technological transitions, sites and storage rooms, and it is customary to save on their illumination. This increases the production safety and comfort of the company’s employees.

Different types and specific models of thermoelectric converters have different characteristics at different values and temperature gradients. To select the most preferable design of a thermoelectric converter, using the ANSYS software environment, the temperature distribution on the surfaces of the transition cones of an experimental heat exchanger intended for installing a system of thermoelectric converters was modeled.

Based on the simulated temperature and actual geometric characteristics of the transition cones of the heat exchanger, the principles of the layout and fastening of the thermoelectric modules on the surface of the transition cones were developed.

For a reasonable choice of materials and design of the elements of the thermoelectric converter, a laboratory setup was designed and manufactured that makes it possible to evaluate the real characteristics of thermoelectric converters in various operating modes. The unit allows the operator to smoothly control the temperature regimes of the heated and cooled transducer plates, accurately measure their electrical characteristics and evaluate the reliability of the design and ease of installation. A number of experiments were carried using the laboratory installation, which made it possible to identify the most promising designs of TEC modules under various temperature conditions in real operation.

The laboratory stage was followed by field measurements of the actual temperature conditions on the surface of the experimental heat exchanger. Based on the measurement results, we decided to use a thermoelectric cell design that was different from the one chosen in the stage of the laboratory research.

After making appropriate changes to the thermoelectric converter module, semi-industrial tests of the developed system were carried out, and the electrical characteristics were recorded.

## 3. Results

### 3.1. Technical Guidance on the Process of Cooling Gases Using An Experimental Prototype of a Hot Gas Cooling Module

The results of the instrumental measurements, which aimed to optimize the parameters of the processes involved in cooling electrolysis gases through an experimental heat exchanger in the interoperation mode of the operation of gas cleaning using a thermoelectric converter, are presented below.

The studies were carried out in conjunction with instrumental measurements of the environmental performance of the RA-550 electrolysers. The studies were carried out on the site of the experimental-industrial electrolysis building of RUSAL Sayanogorsk LLC.

During the period of research, a system for the automatic control and registration of the operating parameters of the experimental heat exchanger was put into operation. The ambient air temperature at the time of the measurements was 4 °C, and the atmospheric pressure during the measurements was 98.1 kPa. The main technological parameters of the RA-550 electrolyzers at the time of the research are presented in Table 1.

### 3.2. Measurements of the Parameters of the Electrolysis Gas Cooling Processes Using the Experimental Heat Exchanger

Investigations of the parameters of the processes involved in cooling electrolysis gases using the experimental heat exchanger were carried out in accordance with [26].

The measurement data were sent to a control cabinet located in the gas cleaning unit. The registration of the incoming values was carried out for every second frequency. The data were recorded on an external storage medium, and the measurement results were displayed graphically.

**Figure 1:**
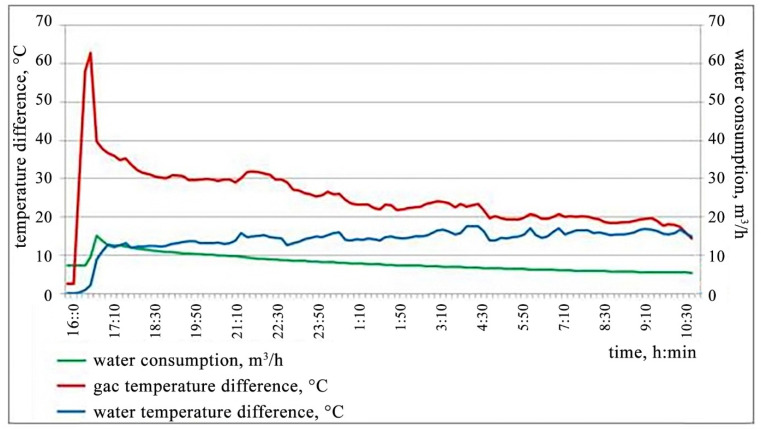
The results of the main parameters of the processes of cooling electrolysis gases using the experimental heat exchanger.

The experimental heat exchanger tests were carried out within 18 h from the start of the installation, and the results are presented in Figure 1.

It follows from Figure 1 that the complete warm-up of the heat exchanger, enabling access to the operating mode, required at least 1 h from the start of the heat exchange process.

The maximum recorded temperature of the process of the gas’ entry into the experimental heat exchanger was 108 °C, which is significantly lower than the critical value (140 °C) and is due to the fact that the operation of the electrolyzers took place in the spring season, as well as the value of the current strength of 530 kA of the experimental electrolyzers compared to the design value (550 kA).

The measured gas flow velocity of 14 m/s corresponds to the design value (more than 10 m/s) that provides a reduction in the mass of the dust deposits on the heat exchange elements of the experimental heat exchanger structure.

It was noted that, during the entire test period, there was a significant decrease in the volume of coolant supplied. This was due to external factors, namely the mode of operation of the plant’s water supply system. As a result, there was a decrease in the efficiency of the heat exchange between the gas and the coolant.

It was determined that a water flow rate of 10 m^3/^h makes it possible to achieve a decrease in the temperature of the exhaust gases at the inlet and outlet of the experimental heat exchanger to 30 °C, while the heating of the coolant does not exceed 15 °C when increasing from the initial value. At a water flow rate of 5 m^3/^h, the gas temperature difference does not exceed 15 °C, which is a low value and does not provide the required heat exchange efficiency. Nevertheless, it was noted that the control of the process control system for the supply of the coolant using the control damper of the flow meter makes it possible to properly control the flow of water.

During the tests, it was found that the outlet manifold pipes on 4 out of 13 heat exchange cassettes have a significantly lower surface temperature (about 15–20 °C), while the temperature of the rest was up to 70–90 °C.

As a result, it was concluded that due to the low water pressure, the design of the nozzles leads to air locks and, as a result, to the formation of “stagnant zones”. To solve this problem, it is necessary to correct the design documentation and make design improvements to the experimental heat exchanger, which contribute to an increase in the efficiency of the heat transfer parameters.

Another disadvantage of the design is the lack of automatic closing of the gates on the gas ducts. At the moments of the initiation and termination of the heat exchange process of the experimental heat exchanger, the flow of electrolysis gases is redirected by manually moving the reducer regulating the gate position in the bypass gas duct, in the duct where the gas enters the experimental heat exchanger and in the duct where the gas leaves the experimental heat exchanger. Taking into account the complexity of the process and the remoteness of the three gates from one another, this circumstance negatively affects the speed of the emergency stop procedure of the experimental heat exchanger in the event of an emergency.

### 3.3. Initial Data for the Manufacturing of a Gas Cooling Module Using a Thermoelectric Converter

The aim of the work was to create a gas cooling module using a thermoelectric converter (TEC) to recover the thermal energy of the hot anode gases of aluminum electrolyzers, together with the production of electrical energy for technological needs, and to reduce the physical volume of the gases prior to further gas cleaning.

According to the terms of reference, the primary conditions for the operation of the treatment gas ducts of the RA-500 electrolyzers were determined. The main gas cleaning parameters are given in Table 2.

The basis for the design of the prototype of the thermoelectric converter was the calculation model “Temperature distribution on the surface of the experimental heat exchanger transition cone in the ANSYS application package”, according to which, at the inlet gas duct of the gas cooling system, the temperature gradient on the surface of the gas duct is in the range of 100–200 °C. Thus, in order to develop a prototype of the thermoelectric converter, it was necessary to determine the material for the thermoelectric module, which could work stably in this temperature range [11].

The section of the experimental heat exchanger inlet gas duct, where we planned the thermoelectric converter to be located, is a cone-shaped pipe made of steel that is 3 mm thick, with a total pipe surface area of 16 m^2^, through which the gas moves at a temperature of about 200 °C. These are the conditions for the stationary mode of operation of this section of the gas duct. The latest research in this area is presented in other works [9,27,28,29].

**Figure 2:**
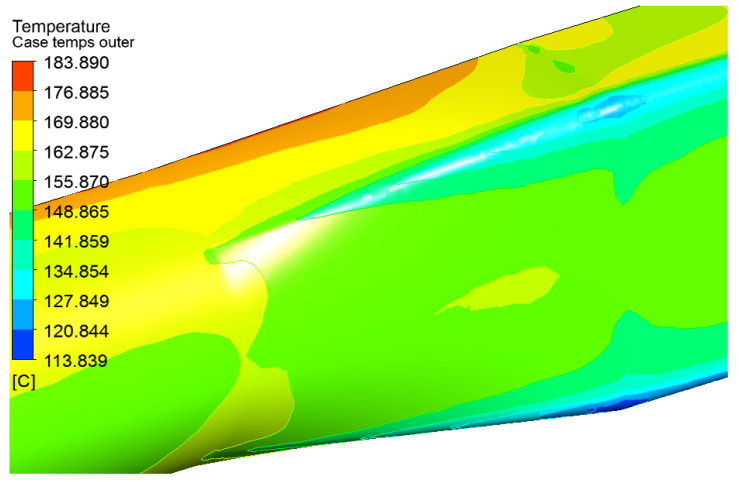
Temperature distribution on the surface of the experimental heat exchanger cone.

Initially, the site of the gas cooling module’s installation was determined based on the section of the gas duct on the segment of the experimental heat exchanger transitional cone, with a surface area of 16 m^2^. These and other data served as the basis for the creation of the calculation model, “Temperature distribution on the surface of the transition cone of the heat exchanger in the ANSYS application package”, according to which the temperature gradient on the surface of the gas duct was in the range of 113–183 °C, while most of the surface area had temperatures in the range of 140–170 °C (Figure 2).

Thus, taking into account the available surface area of the experimental heat exchanger inlet gas duct section (*S_in_*), the area of one thermoelectric converter (*S_TEC_*) and the total power required in accordance with the terms of reference (*P_tot_*), a preliminary calculation of the minimum power of one thermoelectric converter (*P_TEC_*) for the gas cooling module was performed:

- *S_in_* = 16 m^2^;
- *P_tot_* = 8000 W;
- *S_TEC_* = 0.028 m^2^.

In this case, the number of thermoelectric converters (*n*) that can be installed on the entire area of the experimental heat exchanger inlet gas duct section is [30]:

(1) $$n=\frac{S_{in}}{S_{TEC}}=\frac{16}{0.028}=571\;\;pcs$$

In this case, the minimum power of one thermoelectric converter is [30]:

(2) $$P_{TEC}=\frac{P_{tot}}{n}=\frac{8000}{571}=14\;\;W$$

### 3.4. Description of the Module for the Thermoelectric Converter

At present, the main materials used for the production of thermoelectric modules are bismuth, lead, antimony tellurides and their solid solutions; bismuth and antimony selenides; and germanium telluride, samarium monosulfide, gadolinium selenide, stannide and magnesium silicide. For thermometry, semiconductors from a number of metallic alloys of noble and non-noble metals are used.

Due to the fact that all the thermoelectric modules of a new type are currently undergoing a series of experimental studies, they are not freely available for sale, and those that are available are sold at an unreasonably high price, we decided to use thermoelectric modules of the “classical” type. These are a set of series-connected thermocouples. If at least one junction or conductor fails, the entire system will lose its operability. Thus, it is necessary to clearly design systems in accordance with their use and avoid thermal overloads during their operation. The produced thermoelectric modules are formed on glass-ceramic (ceramic) substrates of 60 × 48 or 40 × 40 mm^2^ in size, and the n-type and p-type conductors are created in the form of “pillars” connecting plates. The standard distance between the plates is 2–5 mm. At low operating powers, thermoelectric modules have the highest efficiency among the similar devices, while possessing a relatively low cost and high reliability. The advantages that they have cause a constant increase in the demand for them around the world and the emergence of new areas for their implementation.

**Figure 3:**
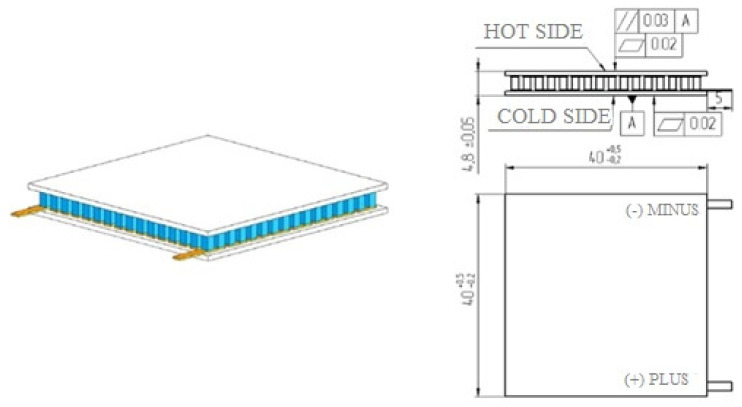
Thermoelectric module for the thermoelectric converter.

Figure 3 shows a thermoelectric module of the “classic” design, which was used to create a prototype of the thermoelectric converter.

The thermoelectric module for the thermoelectric converter has the following technical characteristics:

- Operating temperature up to 200 °C;
- Maximum operating temperature 220 °C;
- Generated power, P (at T_h_ = 200 °C, T_c_ = 30 °C) up to 4.5 W;
- Maximum current 1.36 A;
- Maximum voltage 3.6 V;
- Internal resistance at operating temperature (at 200 °C ± 10%) 3.0 Ohm;
- Material of ceramic plates: Al_2_O_3_ ceramics (VK-96);
- Thermal resistance 2.6 K/W;
- Flatness up to 0.01 mm;
- Parallelism up to 0.01 mm;
- Sealing: epoxy sealant;
- Number of thermoelectric pairs 127;
- The number of thermoelectric elements is 254, and each element has a cross-section of 1.4 mm × 1.4 mm and a height of 2.5 mm;
- Overall dimensions of the module (L × W × H): 40 mm × 40 mm × 4.8 mm.

**Figure 4:**
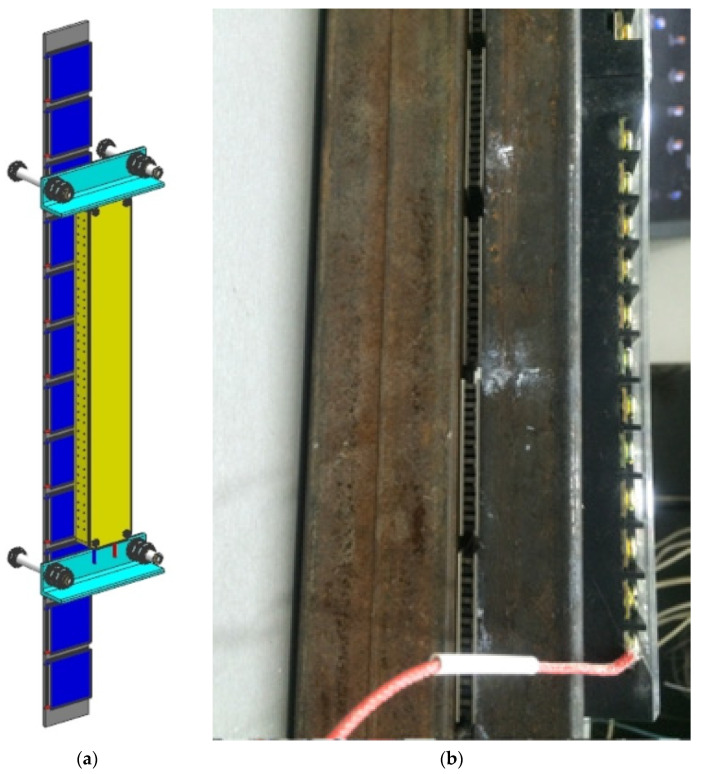
Installation of a heat sink for cooling thermoelectric modules: (**a**) scheme; (**b**) appearance.

One thermoelectric converter includes 12 thermoelectric modules, which are installed on a heat-removing plate (heat removal device) by fixing it with the KPT-8 silicon-organic paste and pressing the cooling radiator with a mounting bracket with bolts that can control the tightening torque (Figure 4). The clamping force of the modules should be at least 12–15 kgf/cm^2^ [31].

**Figure 5:**
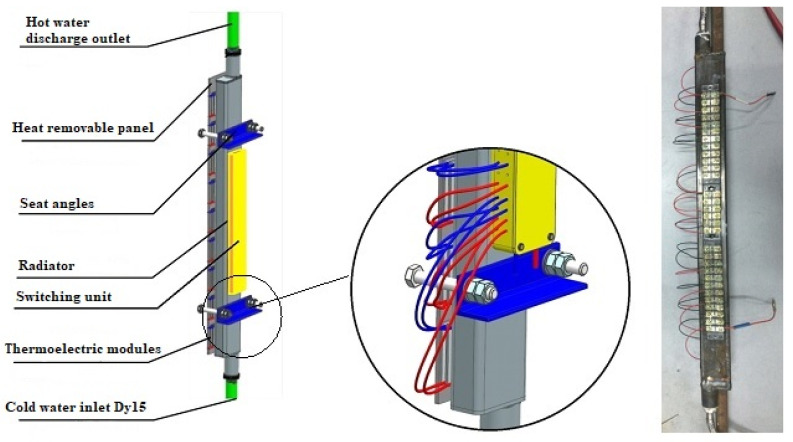
General view and commutation of the thermoelectric modules.

A general view of the thermoelectric converter and electrical connection of the thermoelectric modules is presented in Figure 5.

This kind of heat transfer process is called heat transfer. The basic law of heat transfer is Newton’s law of cooling.

The heat transfer coefficient α determines the amount of heat, measured in Joules (J) and Watts (W), which is transferred from 1 m^2^ of the heat exchange surface to liquid (gas), or vice versa, from liquid (gas) to 1 m^2^ of the heat exchange surface, for 1 s at a temperature difference between the coolant and the heat exchange surface of 1 K. The heat transfer coefficient depends on the following factors:

- The liquid (gas) velocity *ω*, its density *ρ* and viscosity *μ*, that is, variables that determine the mode of flow of the coolant;
- The thermal properties of the coolant (specific heat capacity *c^ᵖ^*, thermal conductivity *λ*), as well as the volume expansion coefficient *β*;
- The geometric parameters, including the shape and determining dimensions of the wall (for the pipes, their length *L* and diameter *d*), the wall roughness *ε*;
- The heat transfer coefficient *k*.

In this case, the heat transfer coefficient is [30]:

(3) $$\alpha =f\left({\omega ,\;\rho ,\;\mu ,\;c}^{P},\;\lambda ,\;\beta ,\;d,\;L,\;\varepsilon ,\;k\right)$$

From this general dependence, we can conclude that the simplicity of Newton’s cooling law equation is apparent.

On the other hand, in a number of industrial systems, heat removal, so as to obtain a liquid medium, is not a problem. Moreover, in some cases (for example, in heating boilers), it is the only acceptable solution.

Following the above methodology, for such designs, the modules must have a thermal resistance of more than 2 K/W.

An important factor determining the choice of module is its location. Often, it makes its own adjustments to the final version of the design.

It is possible that the material from which the radiator is made has a porous internal structure and the presence of a large number of impurities in the composition, which could significantly affect the thermal conductivity.

**Figure 6:**
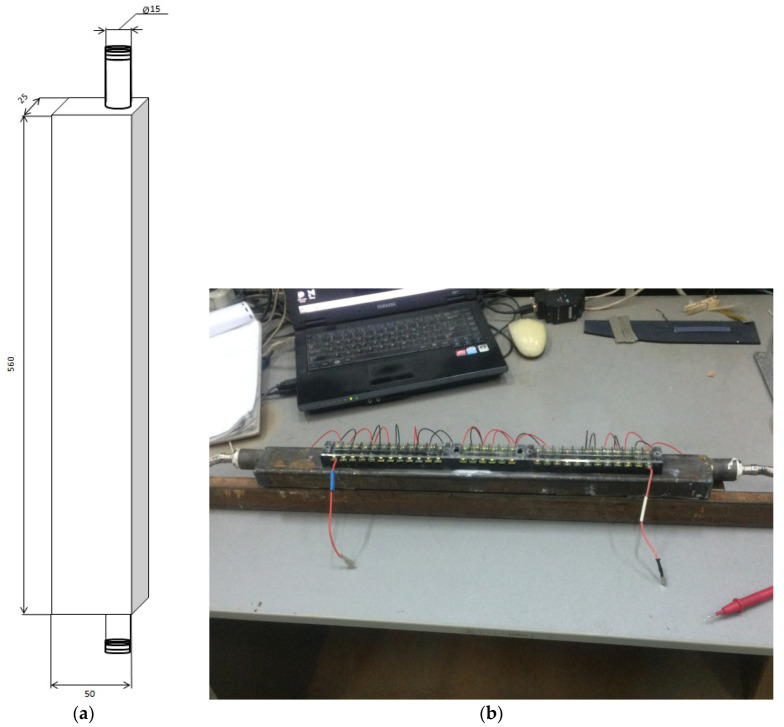
Steel water cooling radiator for the thermoelectric converter: (**a**) drawing; (**b**) appearance.

For this reason, we created samples of steel water radiators (Figure 6). In addition, these radiators were pressure-tested at 10 bar.

Additionally, pressurization tests at a pressure of 10 bar were carried out on these radiators.

### 3.5. Description of the Gas Cooling Module Using the Thermoelectric Converter

**Figure 7:**
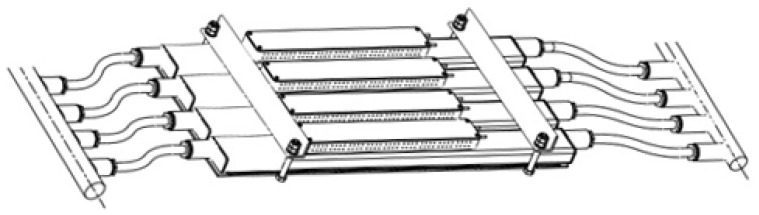
Gas cooling module using the thermoelectric converter.

The gas cooling module is a structure consisting of four blocks of thermoelectric converters interconnected by flexible high-temperature piping (Figure 7). The module is designed for installation on gas ducts of various diameters.

The main functions of the gas cooling module using the thermoelectric converter are:

- The decrease in the temperature and physical volume of the gases being cleaned;
- The conversion of the thermal energy of the cleaned gases into electrical energy;
- The power supply of the energy-consuming devices located in the process area.

**Figure 8:**
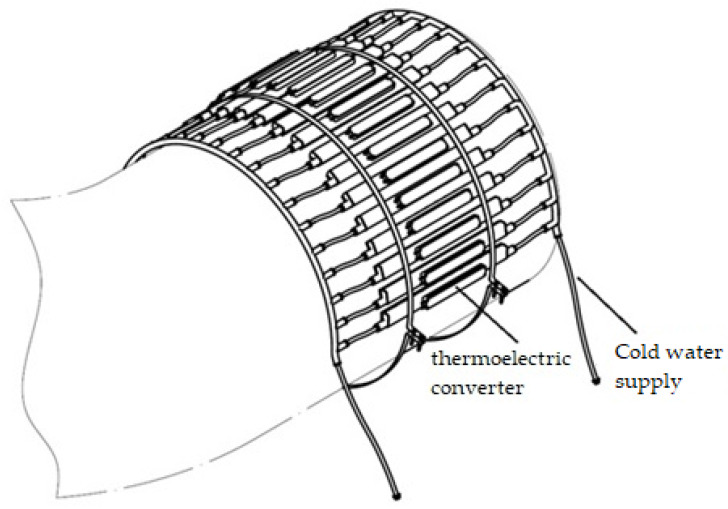
Layout option for a gas cooling system consisting of several thermoelectric converter modules.

The modules for cooling gases with the help of a thermoelectric converter can, if necessary, be combined with each other, forming the so-called gas cooling system that is operated using the thermoelectric converter modules (Figure 8).

**Figure 9:**
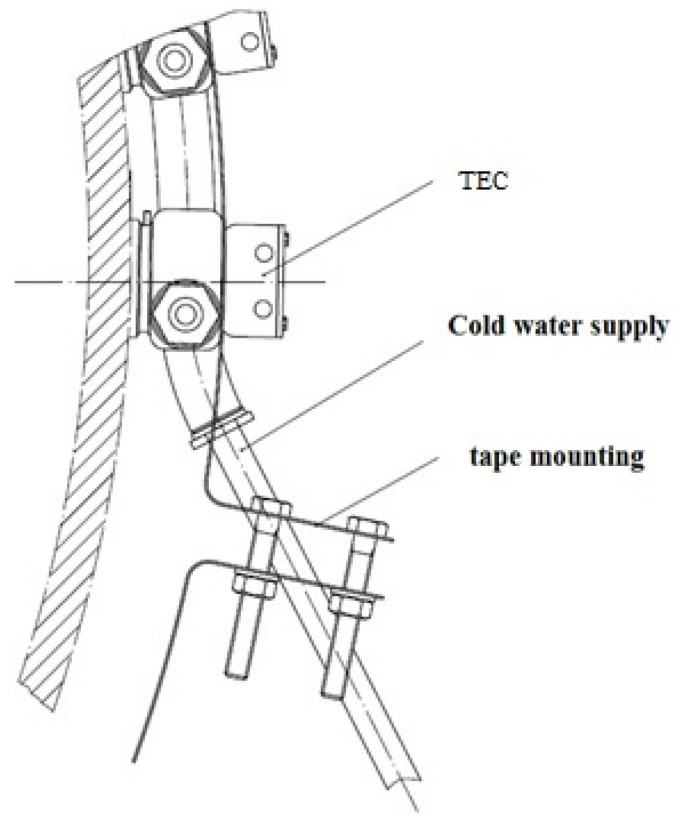
Method of fastening the gas cooling system to the flue pipe.

To mount a system of cooling modules, a perforated fastening tape is used, which is tightened with a bolted connection, as shown in Figure 9.

The principle of operation of the gas cooling module using the thermoelectric converter is as follows. The gas moves through the heat exchanger to the gas cleaning system. To cool the module, water provided for technological needs is used. The module converts the created temperature gradient from the hot gas and coolant into electricity, which is then transferred to the load elements.

### 3.6. Description of the Thermoelectric Converters for the Gas Cooling Module

Since the gas cooling module has a design of four blocks of thermoelectric converters, we will consider the elements that form one block of thermoelectric converter in detail.

The main elements of the thermoelectric converter block are:

(a) The heat sink plate (heat sink);
(b) The thermoelectric modules;
(c) The cooling radiator;
(d) The switching unit.

The heat-removing plate (heat-removing device) ensures the efficient heat transfer from the flue wall to the thermoelectric modules.

**Figure 10:**
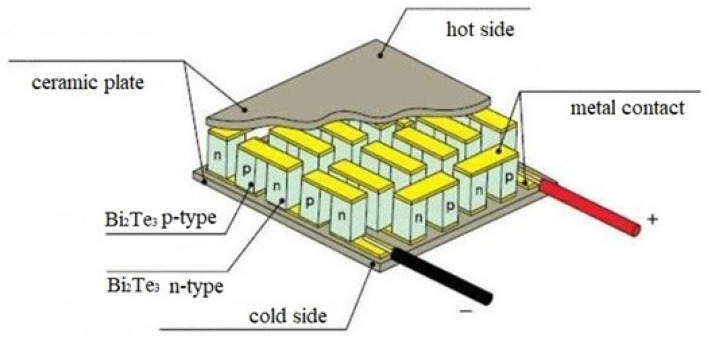
The structure of the thermoelectric module.

Thermoelectric modules are semiconductor generators that generate an electric current due to the temperature difference between its opposite surfaces (Figure 10).

In recent years, bismuth telluride Bi_2_Te_3_, with a thermoelectromotive force coefficient of 230 μV/K, and some of its solid solutions with isomorphic compounds, which are used to increase the mobility of electricity carriers and reduce the thermal conductivity of the crystal lattice, have received the most frequent use in the manufacturing of thermoelectric modules. The latter are bismuth selenine Bi_2_Se_3_, with a thermoelectromotive force coefficient of 300 μV/K, and antimony tellurium Sb_2_Te_3_, with a thermoelectromotive force coefficient of 100 μV/K [32,33,34]. There are also innovative solutions using PbTe, GeBiTe and Bi_0.5_Sb_1.5_Te_3_ [35,36,37,38].

An analysis of the technical characteristics and cost of thermoelectric modules showed that, to solve this problem, it is expedient to use modules based on bismuth telluride Bi_2_Te_3_. To date, there is a large selection of such modules, which are manufactured both in series (“classic”) and for solving specific problems where the use of classical modules is not possible, or with the use of new techniques and technologies for the manufacturing of modules.

The distance between the plates is 3.5 mm. Inside one thermoelectric converter block, there are 12 thermoelectric modules based on bismuth telluride Bi_2_Te_3_, with a thermoelectric power factor of 230 μV/K.

The technical characteristics of the thermoelectric module for the thermoelectric converter unit are presented in Table 3.

The conversion efficiency of thermoelectric modules depends on many factors, primarily on the quality of the contact between all the heat transfer elements of the thermoelectric converter unit.

### 3.7. Description of the Cooling Radiator for the Gas Cooling Module

For the gas cooling module, a liquid cooling radiator is used, which runs on process water.

**Figure 11:**
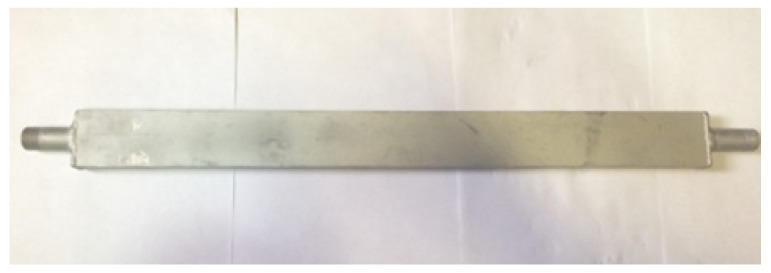
Radiator of the thermoelectric converter unit used in the anode gas cooling module.

The radiator is a hollow tube formed of aluminum alloy of grade AD31T1 (increased corrosion resistance) with a rectangular section of 50 × 20 mm and a wall thickness of 2 mm. Outside the radiator, there are inlet and outlet pipes with a diameter of Dy = 15 mm (3/4″) for the connection to the water supply system and ensuring the circulation of the coolant. The cooling radiator is directly attached to the planes of the thermoelectric modules (ceramic plates) to create effective cooling by heat transfer and is fixed with a hot-melt glue brand, AlSil_5_, to create a heat-conducting layer. Additionally, it is possible to use the thermal paste brand KPT-8 as a temporary fixation and then fix it mechanically. These brands are relatively common and have one of the best thermal insulation performances. Figure 11 shows a heatsink of a thermoelectric converter unit used in a cooling module for anode gases.

**Figure 12:**
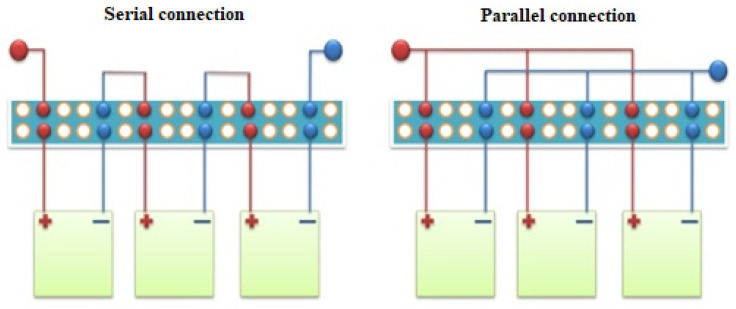
Serial and parallel connections of the thermoelectric modules in the thermoelectric converter switching unit of the gas cooling module.

The switching unit is intended for the connection of thermoelectric modules of the thermoelectric converter unit. The switching unit is located on the side of the cooling radiator of the thermoelectric converter unit. Thus, to obtain the maximum voltage, for example, the modules are connected in series (Figure 12).

The switching unit has output terminals to create a further connection with the subsequent thermoelectric converter units.

### 3.8. Laboratory Investigations Regarding the Reasonable Selection of the Material and Design of the Thermoelectric Converter

For the purpose of a preliminary assessment of the efficiency of the thermoelectric converters, a model installation was designed. The installation allows for the simulation of various modes of cooling and heating of the TEC, and it is also equipped with measuring equipment. The installation has the following components:

(a) Imitation of a flue. The gas duct is formed by a metal profile pipe, through which air at the required temperature is supplied by means of an industrial hair dryer with a maximum outlet air temperature of more than 600 °C;
(b) The TEC unit consisting of the four tested TEC elements, placed between the gas duct simulation unit and the cooling unit.
(c) The cooling unit. This is a profiled aluminum radiator, the end outlets of which are connected through a flow meter and a thermometer to a closed water circulation system.
(d) Optional equipment. Placement table, fastening system, etc.

The measuring equipment of the laboratory setup consists of the following components:

(a) Thermal anemometer 405-v1;
(b) SIMATIC SM-1231 controller;
(c) Set of thermocouples;
(d) Digital multimeter Fluke 77 IV;
(e) Digital thermometer TESTO 104-IR.

Technically, the laboratory setup is a single module of the thermoelectric converter described in the previous sections.

After creating a prototype for the testing, a laboratory bench was used, which, using the analog temperature sensors attached to the surface of the thermoelectric converter and cooled surfaces, determines parameters such as the current strength and voltage of the thermoelectric converter. Furthermore, the obtained data were processed using the SIMATIC SM-1231 controller.

To improve the purity of the experiment, an increase in the contact surface was carried out in order to improve the thermal contact between the surfaces of the thermoelectric converter and the cooled surface. To increase it, heat-conducting paste KPT-8 was used. Resistance thermometers have a flexible shape and are fixed to specially prepared areas of the thermoelectric converter cooling module [31].

The use of temperature sensors and resistance thermometers made it possible to determine the heat transfer from the liquid to the thermoelectric converter.

To optimize the parameters, three types of thermoelectric modules with different technical characteristics were acquired, the designs of which enable heat removal through liquid cooling, performed in the gas cooling module:

(a) TGM 127-1.4-2.5 (previously used in the thermoelectric converter block);
(b) TGM 199-1.4-0.8;
(c) TGM 127-1.4-1.2.

**Figure 13:**
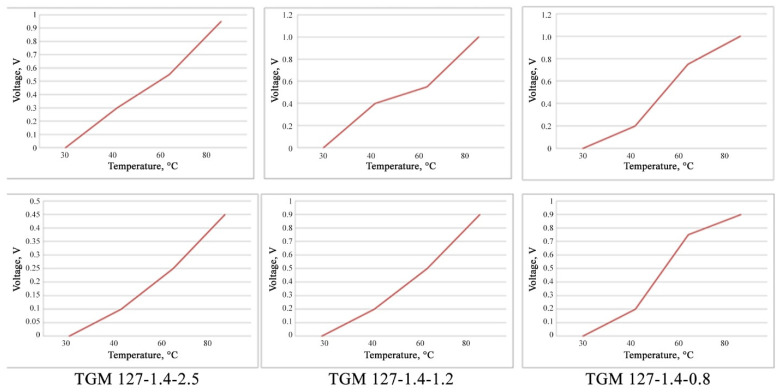
Dependences of the output indicators of the thermoelectric generators: TGM 127-1.4-2.5; TGM 199-1.4-0.8; TGM 127-1.4-1.2.

Graphs showing the dependences of the output indicators of the TEC modules on the temperatures obtained in the laboratory conditions are shown in Figure 13.

The laboratory tests showed that, at temperatures of the hot side of the experimental gas duct model of about 80 °C, the model TGM 127-1.4-1.2 should be used, which has better characteristics at low temperature gradients of the cold and hot sides. The parameters of the dependence of the output power on the temperature of the heat transfer surface for the three types of thermoelectric modules are presented in Table 4.

### 3.9. Calculation of the Output Power of the Gas Cooling Module

The laboratory tests of the thermoelectric converter unit showed its stable operation in the steady state heat exchange mode. Additionally, the electrical output parameters of one thermoelectric converter unit were determined in various operating modes. Based on the terms of reference, the optimal temperature regimes for the operation of the gas ducts and coolant were selected. The average temperature of the hot surface of the heat-removing plate was chosen according to the calculation model of the temperature distribution on the surface of the experimental heat exchanger cone.

A preliminary calculation of the output power of the gas cooling module was performed. According to the calculations, the module provided an output power of 79 W at a temperature of the hot surface of the heat-removing plate of 150 °C, and the temperature of the coolant (water) that circulated in the cooling radiator of the thermoelectric converter unit was 28 °C.

An increase in the temperature of the hot surface of the heat-removing plate to a temperature of 200 °C made it possible to increase the output power of the thermoelectric converter unit. In this case, the output power of the module was 97 W.

A decrease in the temperature of the hot surface of the heat-removing plate significantly reduced the performance of the thermoelectric converter unit. Thus, at a temperature of the hot surface of the heat-removing plate of 100 °C, the power of the module is 38 W (Table 5).

Thus, the efficiency of the gas cooling module is significantly affected by both the temperature of the heat transfer surface and the temperature difference created between the cooling radiators of the thermoelectric converter blocks and the hot heat transfer surface.

The area occupied by the gas cooling module of the four thermoelectric converter blocks, taking into account the inter-block distances and inlet water nozzles, is 0.44 m^2^. Under the operating conditions of a four-block gas cooling module with an output power of 79 W at a given gas duct wall temperature, at least 13 modules are required to produce 1 kW of electricity. The area required for their placement is 5.72 m^2^.

### 3.10. The Results of the Measurements of the Temperature Regimes for the Operation of the Gas Ducts in the Autumn Period of the Year

**Figure 14:**
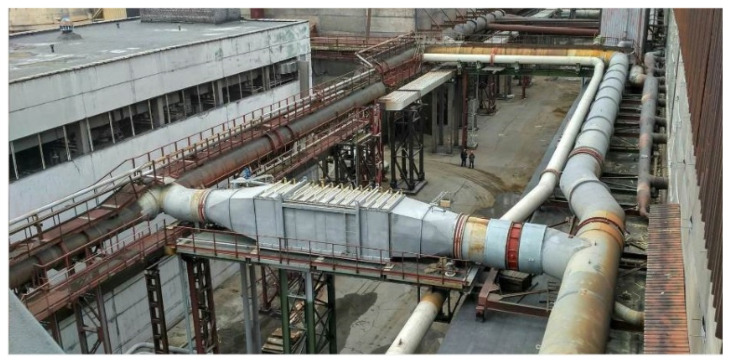
Gas ducts derived from the electrolytic cells of the RA400, RA500 series.

Measurements carried out in October at the site of the pilot electrolysis building of RUSAL Sayanogorsk LLC showed that the calculated temperature distribution model, on the basis of which the gas cooling module was developed, does not coincide with the actual temperature values of the surfaces of the walls of the gas ducts (Figure 14). Thus, the average temperature of the outer walls of the gas flues, according to the calculation model, is around 150 °C. The field measurements showed that, in autumn, the temperature of the walls of the gas duct does not exceed 85 °C. It was determined that the use of the thermoelectric modules, TGM 127-1.4-2.5, which are part of the thermoelectric converter unit and perform the function of heat-electric conversion, is possible only in the summer season, when the temperature of the surfaces of the gas duct is in the range of 150–200 °C. In the autumn-winter period, the gas cooling module is not able to provide the required performance.

In this regard, the optimization of the existing operation parameters of the gas cooling module using the thermoelectric converter was carried out based on an experimental model of a gas duct using a laboratory stand. The studies were carried out whilst taking into account the temperature conditions of the gas ducts and the temperature of the coolant of the technological line, measured in October 2017. As part of this work, it was determined that the optimization of the parameters is possible when using thermoelectric modules of different types.

Based on the foregoing observations, for the manufacturing of the thermoelectric converter block of the gas cooling module, thermoelectric modules of the TGM 127-1.4-1.2 type were used. The block was tested directly on the gas duct section to determine the compliance of the output parameters with the obtained laboratory data.

### 3.11. The Results of the Measurements of the Temperature Regimes for the Operation of the Gas Ducts in the Spring Period of the Year

The tests were carried out in April at the site of the pilot electrolysis building of RUSAL Sayanogorsk LLC. The ambient temperature at the time of testing was +9 °C. The test procedure was carried out according to the same principle as before.

To register the measured indicators, we applied the measuring instruments which were used in the process of the previous stages of this work.

**Figure 15:**
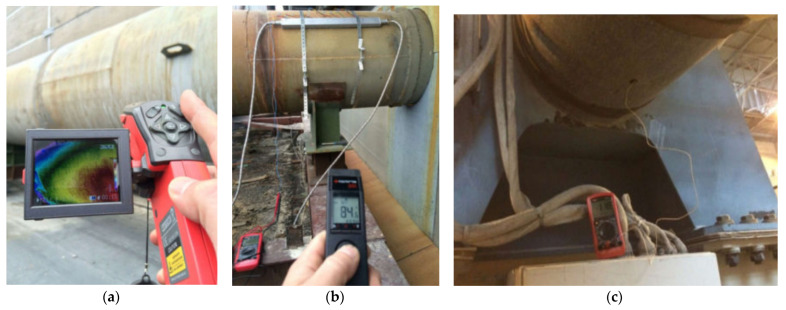
Measurement of the indicators of the temperature values of the walls of the gas ducts with the measuring tools: (**a**) UNI-X thermal imager; (**b**) digital pyrometer Optris MS Plus; (**c**) UNI-X digital multimeter.

The temperature values of the walls of the gas ducts were determined using three devices to eliminate the possibility of measurement errors: a UNI-X thermal imager, a digital thermometer “TK-5.04” with an immersion and contact thermocouple, and a digital pyrometer, Optris MS Plus (Figure 15a,b). The output voltage and current indicators were determined using a UNI-X digital multimeter (Figure 15c).

The first step was to determine the temperature at the exit point of the anode gases from the cell, which was 125 °C, exceeding the previously measured temperature by 7 °C. However, this temperature does not provide the necessary thermal threshold to ensure the effective heating of the gas duct wall and further heat transfer to the gas cooling module in order to provide an effective heat load.

**Figure 16:**
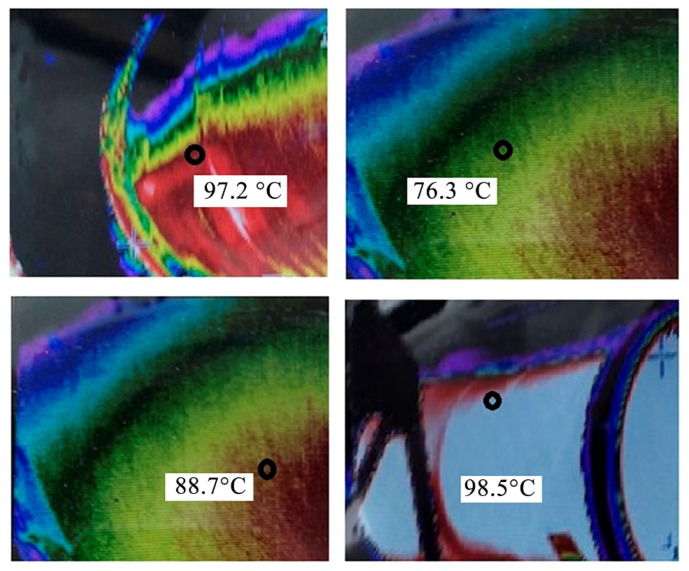
Temperatures of the surface of the walls of the gas duct.

Furthermore, we performed an analysis of the temperature indicators of the walls of the gas ducts based on the electrolyzers of the RA-500 series along the entire perimeter, as well as the temperature indicators of the walls of the gas ducts of the RA-400 series, which showed that the temperature distribution was in the range from 75 to 100 °C, depending on the location of the measuring points and distance from the electrolysis building. The highest temperatures were determined directly in the initial zones of the ducts, which indicated large heat losses due to the large area of the heat transfer surfaces of the gas ducts, as well as the uneven flow inside the ducts themselves. The maximum temperature, as before, was recorded in a small area (its upper part) at the starting point of the gas duct based on the RA-500 electrolyzers and amounted to 98.5 °C. Therefore, for the measurements, the installation location of the thermoelectric converter block of the cooling module was determined to be in this place (Figure 16).

### 3.12. Description of Semi-Industrial Tests of the Thermoelectric Converter Unit

**Figure 17:**
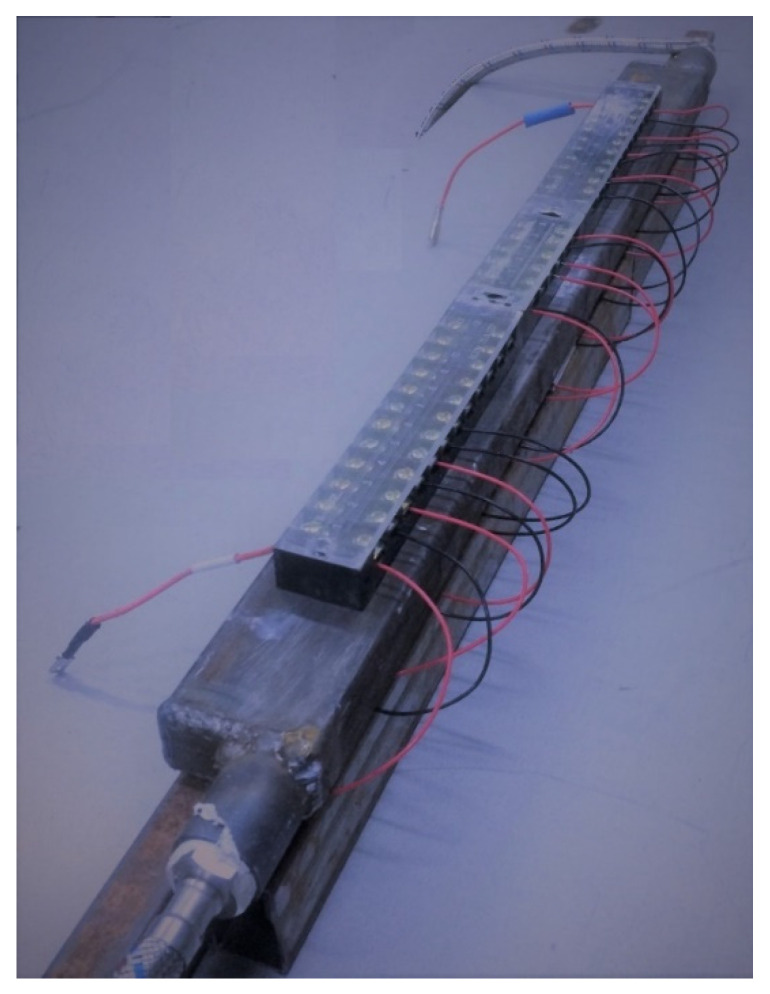
Unit of the thermoelectric converter of the gas cooling module.

As the object of the test, a thermoelectric converter block was used for a gas cooling module with 12 TGM-127-1.4-1.2 thermoelectric generators, which were used in the manufacturing of a thermoelectric converter block after its optimization for lower temperatures (Figure 17).

**Figure 18:**
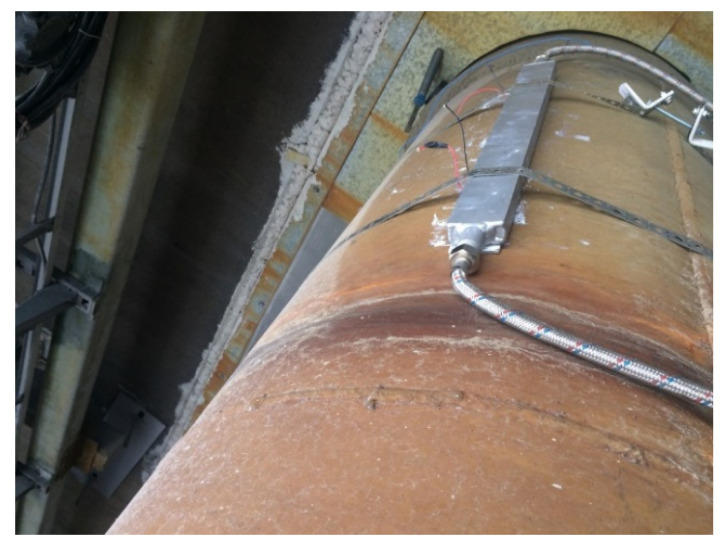
Unit of the thermoelectric converter, installed on the gas duct.

The unit of the thermoelectric converter was fixed to the gas duct using perforated fastening tape, enabling the possibility of tightening (Figure 18). As a source of water supply, a mobile point was used to supply coolant (water) to the cooling radiator of the thermoelectric converter unit. For this purpose, we used a 125 L tank and a circulation pump, which was attached to the outlet pipe at the bottom of the tank (closed system).

The water temperature was measured using an immersion thermocouple connected to a TK-5.04 digital thermometer. The temperature of the cooling liquid (water) corresponded to the temperature of the water in the process water supply line system and was 21 °C. Thus, the difference between the temperatures of the hot side of the gas duct wall at the point of the installation of the thermoelectric converter unit and the temperature of the radiator coolant was 77.5 °C.

**Figure 19:**
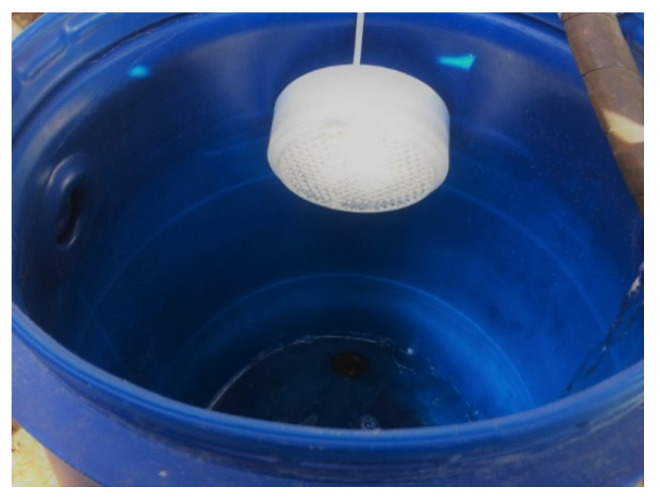
The operation of the LED lamp due to the converted thermal energy of the anode gases.

The connection of the radiator of the thermoelectric converter unit with the mobile water supply point was created using a flexible plumbing connection. Thermal grease KPT-8 was used as a heat-insulating layer between the thermoelectric generators, heat-removing plate and cooling radiator. The load element was an LED flashlight with a power of 6 W, 12V (Figure 19).

Thus, the difference between the temperatures of the hot side of the gas duct wall at the point of the installation of the thermoelectric converter unit and the temperature of the radiator coolant was 77.5 °C, which exceeded the previous figure by 17.5 °C (in autumn, the temperature difference did not exceed 60 °C).

Measurements of the output indicators of the thermoelectric converter block were carried out in the following sequence:

- Setting up a water supply source (circulation pump) and monitoring the temperature of the coolant;
- Temperature stabilization of the heat-removing elements of the thermoelectric converter block (5 min);
- Stabilization of the heat exchange mode (10 min);
- Fixing the results based on the measurements of the output indicators of the voltage and current.

The measurement results showed that the data obtained as a result of the laboratory tests of the output indicators of the thermoelectric converter block of the gas cooling module are quite similar to the readings of the field tests. At a temperature difference of 75–80 °C between the wall surface of the gas duct section and the coolant, the voltage indicators of the thermoelectric converter block are in the range of 15–17 V, and the current indicator is in the range of 0.5–0.6 A.

During the testing period, the LED flashlight, shown in Figure 18, worked stably, without attenuation.

Despite the fact that the temperature of the gas duct wall is not optimal for the efficient operation of the thermoelectric converter unit (with a temperature gradient of about 150–170 °C, the power of one unit reached 24 W), this makes it possible to use the electrolysis gas cooling system using the thermoelectric converter as an additional measure for the utilization of part of the thermal energy of the electrolysis gases and to convert thermal energy into electricity for production needs.

The total power of the thermoelectric converter block of the gas cooling module during the full-scale tests is presented in Table 6.

## 4. Discussion

Based on the results of our studies of the parameters of the processes involved in cooling electrolysis gases, it was determined that the experimental heat exchanger provided a decrease in the temperature of the process gas at the outlet from 30 to 15 °C, depending on the flow rate of the water supplied for the cooling in the range of 10 to 4 m^3^/h.

During the tests, it was noted that:

1. Design improvements are required to improve the efficiency of the experimental heat exchanger due to factors such as the formation of “stagnant zones” owing to the low water pressure.
2. There is an uncontrollable decrease in the water consumption due to a decrease in the pressure drop in the water supply system of the plant. In the future, it is necessary to ensure interaction with the relevant services of the plant.
3. To redirect the gas flow, it was necessary to open the three gates manually, which is a laborious and time-consuming process. To ensure the trouble-free operation of the experimental heat exchanger, we recommend installing automatic gates.
4. During the additional tests, an error was found in the process of obtaining data from the process control system through a thermocouple measuring the gas temperature at the outlet of the experimental heat exchanger. This requires the involvement of the employees of the contractor involved in the installation of the process control system to check the quality of the installation.

It should be assumed that, with an increase in the coolant flow rate over 10 m^3^/h and the introduction of structural changes by moving the outlet manifold above the level of the branch pipes, a significant increase in the cooling of the exhaust gases will occur, which is especially important in the summer season for the experimental electrolyzers. To obtain more accurate data on the thermal and aerodynamic characteristics of the experimental heat exchanger after its optimization, it is necessary to conduct repeated studies of the heat transfer parameters.

## 5. Conclusions

In the course of the work, the following results were achieved:

1. The design of the gas cooling system, with the help of the thermoelectric converter, was developed, consisting of blocks of thermoelectric converters interconnected by flexible high-temperature piping.
2. Based on the test results, the zone of the maximum temperatures in the section of the gas duct recommended for the installation of a gas cooling module using a thermoelectric converter was determined.
3. The technology for cooling gases with the help of the thermoelectric converter was tested on the site located in front of the experimental heat exchanger. An assessment of the efficiency of the conversion of heat into electrical energy was performed using the design of the thermoelectric converter unit, based on the thermoelectric modules TGM 127-1.4-1.2.
4. It was determined that the device is capable of generating electricity stably for production needs. The data obtained showed that, at a temperature difference of 75 ÷ 80 °C between the wall surface of the gas duct section and the coolant, the power of one thermoelectric converter block of the gas cooling system reaches 9 W.
5. When operating a system that consists of 25 thermoelectric converter blocks based on TGM 127-1.4-1.2 modules encircling the circumference of the gas duct with a surface temperature of 90 °C and a diameter of 800 mm, the maximum generated power can be over 200 W. When using LED lamps with a power consumption of 6–8 W, the conversion of thermal energy into electrical energy will meet the technological needs for the artificial lighting of the production site.
6. As a result of the studies that we carried out, it was determined that, for a more complete and high-quality utilization of the heat of exhaust gases, it is worth directing part of the energy to preheat the baked anodes and using more modern TECs to increase the efficiency of the energy conversion.

In the future, we plan to carry out work on the integrated use of the waste gas heat, consisting of three areas: thermoelectric conversion, the heat exchanger and the preheating of anodes. In the complex development process, we plan to use bulk and film TECs of a new generation, using Sr_0.9_La_0.1_TiO_3_ and Pb-Cd-Te.

## Figures and Tables

**Table 1:** Parameters of the dependence of the output power on the temperature of the heat transfer surface of the flue.

Parameter Name	Unit	Actual Value	Design Value
Current strength	kA	530	550
Process electricity consumption	kWh/t Al	13,050	12,900
Current output	%	94.5	95.0
Medium voltage	AT	4.11	4.118
Daily performance	kg	4040	4213.4
Electrolyte temperature	°C	962	-
Cryolite ratio	USD units	2.2	-
Consumption of baked anodes	kg/t Al	525	410
Alumina consumption	kg/t Al	1915	1918
Aluminum fluoride consumption	kg/t Al	12	18.2

**Table 2:** Operating parameters of the cleaning gas ducts of the RA-500 electrolyzer.

Parameter Name	Unit	Value
The volume of exhaust gases (from 8 electrolyzers)	nm^3/^h	75,000
Initial gas temperature	°C	up to 200
Final gas temperature	°C	no more than 140
Gas speed	m/s	not less than 10
Flue wall material	-	Steel
Wall thickness	mm	3
Water temperature for technological needs	°C	30

**Table 3:** Parameters of the operation of the thermoelectric converter modules.

Parameter Name	Unit	Value
Working temperature	°C	up to 200
Maximum operating temperature	°C	220
Generated power at 30 °C coolant temperature and 200 °C flue wall temperature	W	up to 4.5
Maximum current	A	1.36
Maximum voltage	V	3.6
Internal resistance at operating temperature 200 °C	Ohm	3.0
Number of thermoelectric pairs	pcs	127
Number of thermoelectric elements	pcs	254
Cross-section of thermoelectric element (L × W × H)	mm	1.4 × 1.4 × 2.5
Overall dimensions of the module (L × W × H)	mm	40 × 40 × 4.8

**Table 4:** Parameters of the dependence of the output power on the temperature of the heat transfer surface for the 3 types of thermoelectric modules.

Generator Type Name	Temperature, °C	Power, W
TGM 127-1.4-2.5	40	0.03
TGM 127-1.4-2.5	60	0.125
TGM 127-1.4-2.5	80	0.45
TGM 199-1.4-0.8	40	0.04
TGM 199-1.4-0.8	60	0.56
TGM 199-1.4-0.8	80	0.9
TGM 127-1.4-1.2	40	0.08
TGM 127-1.4-1.2	60	0.275
TGM 127-1.4-1.2	80	0.9

**Table 5:** Preliminary calculation of the output power of the gas cooling module.

Flue Wall Temperature, °C	Coolant Temperature, °C	Output Power of the Gas Cooling Module, W
150	28	79
200	28	97
100	28	38

**Table 6:** Parameters of the dependence of the output power on the temperature of the heat transfer surface of the flue.

Generator Type Name	Flue Wall Temperature, °C	Coolant Temperature, °C	Power, W
TGM 127-1.4-1.2	98.5	21	9.38
TGM 127-1.4-1.2	92.3	21	8.77
TGM 127-1.4-1.2	96.6	21	9.04

## Data Availability

The data presented in this study are available from the corresponding authors upon reasonable request.
